# Nucleic acid separations using superficially porous silica particles

**DOI:** 10.1016/j.chroma.2016.02.057

**Published:** 2016-04-01

**Authors:** Elizabeth D. Close, Alison O. Nwokeoji, Dafydd Milton, Ken Cook, Darsha M. Hindocha, Elliot C. Hook, Helen Wood, Mark J. Dickman

**Affiliations:** a Department of Chemical and Biological Engineering, ChELSI Institute, University of Sheffield, Mappin Street, Sheffield S1 3JD, UK; b Thermo Fisher Scientific, Stafford House, Boundary Way, Hemel Hempstead HP2 7GE, UK; c GlaxoSmithKline, Medicines Research Centre, Gunnels Wood Road, Stevenage, Hertfordshire SG1 2NY, UK

**Keywords:** Ion pair reverse-phase chromatography, Nucleic acids, RNA, Oligonucleotides

## Abstract

Ion pair reverse-phase liquid chromatography has been widely employed for nucleic acid separations. A wide range of alternative stationary phases have been utilised in conjunction with ion pair reverse-phase chromatography, including totally porous particles, non-porous particles, macroporous particles and monolithic stationary phases. In this study we have utilised superficially porous silica particles in conjunction with ion pair reverse-phase liquid chromatography for the analysis of nucleic acids. We have investigated a range of different pore-sizes and phases for the analysis of a diverse range of nucleic acids including oligonucleotides, oligoribonucleotides, phosphorothioate oligonucleotides and high molecular weight dsDNA and RNA. The pore size of the superficially porous silica particles was shown to significantly affect the resolution of the nucleic acids. Optimum separations of small oligonucleotides such as those generated in RNase mapping experiments were obtained with 80 Å pore sizes and can readily be interfaced with mass spectrometry analysis. Improved resolution of larger oligonucleotides (>19 mers) was observed with pore sizes of 150 Å. The optimum resolution for larger dsDNA/RNA molecules was achieved using superficially porous silica particles with pore sizes of 400 Å. Furthermore, we have utilised 150 Å pore size solid-core particles to separate typical impurities of a fully phosphorothioated oligonucleotide, which are often generated in the synthesis of this important class of therapeutic oligonucleotide.

## Highlights

• Superficially porous silica particles enable high resolution separation of nucleic acids.
• The pore size of the C_18_ superficially porous silica particles significantly effects resolution.
• Optimum separations of small oligonucleotides obtained with 80 Å pore sizes.
• Optimum resolution of oligonucleotides (>19 mers) was observed with pore sizes of 150 Å.
• Improved resolution of larger dsDNA/RNA molecules was achieved with pore sizes of 400 Å

## 1 Introduction

### 1.1 Ion pair reverse-phase chromatography

Ion pair reverse-phase chromatography (IP RP HPLC) has been widely employed for nucleic acid separations [1], [2], [3]. A wide range of reverse-phase stationary phases have been utilised for IP RP HPLC including traditional totally porous C_18_ particles (silica and polymeric stationary phases) [1], [2], [3], [4], [5]. However, slow mass transfer of oligonucleotides in the stationary phase limits the application of these traditional C_18_ based stationary phases [6], [7]. The development of 2 μm, C_18_ surface, non-porous polymeric columns, in conjunction with IP RP HPLC offers significant advantages for the analysis of nucleic acids [8], [9], [10], [11]. Rapid, high resolution separation of nucleic acids has been achieved using these non-porous polymeric media. The highly mono-disperse nature of the particles results in the minimisation of the diffusion pathways and therefore provides high resolution with rapid analysis times [1], [2], [3]. Furthermore, the column media are robust, being resistant to a broad range of temperatures and pH. This has led to a wide variety of developments in the analysis of nucleic acids using IP RP HPLC, including the sequence-independent sizing of duplex DNA [9], the analysis of oligonucleotides and the separation and purification of single-stranded (ss) DNA [10], [12]. However, a caveat associated with these non-porous particles includes low capacity owing to the limited surface area for interaction with the analyte.

The application of non-porous polymeric stationary phases has also been extended to analyse larger RNA molecules in conjunction with IP RP HPLC with high resolution, similar to that achieved for larger dsDNA molecules and was demonstrated in the development of an assay for group I intron ribozyme activity [13]. Further studies have utilised the rapid high resolution separation of RNA to analyse a wide range of RNA transcripts and biological RNAs including ribosomal RNA, mRNA and siRNAs [14], [15], [16], [17]. More recently, macro-porous PS-DVB resins with relatively large pore sizes have been utilised for RNA separations [18]. Ultra-performance liquid chromatography (UPLC) approaches using 1.7 μm C_18_ particles have also been employed to improve speed and resolution for short oligoribonucleotides, oligonucleotide therapeutics and the sequencing of synthetic oligoribonucleotides in conjunction with LC–MS analysis [19], [20], [21]. In addition to traditional particular stationary phases, IP RP HPLC in conjunction with a wide range of monolithic stationary phases have also successfully been applied to the high resolution separation of DNA [22] and RNA [16], [23]. Monolithic, poly(styrene-divinylbenzene)-based capillary columns have been utilised for nucleic acid separations interfaced to mass spectrometry in conjunction with MS-compatible mobile phases in a number of different applications [24], [25], [26], [27].

Columns comprising superficially porous particles (SPPs), also termed core–shell particles, solid-core particles and porous-shell particles have been utilised in wide range of studies to analyse biomolecules, demonstrating significant advantages over traditional totally porous particles [28], [29], [30]. The advantages of core–shell particle columns for rapid HPLC analysis of biomolecules including proteins and nucleic acids has been demonstrated, attributing this improved performance to superior mass transfer kinetics [28], [29]. More recently Biba et al., studied the performance of a number of core–shell particle columns in conjunction with IP RP HPLC for the analysis of oligonucleotides [31], [32].

In this study we have utilised superficially porous silica particles (Accucore columns, Thermo Fisher) in conjunction with IP RP HPLC for the analysis of nucleic acids. The Accucore columns contain 2.6 μm solid-core particles, with a porous layer of 0.5 μm with an average particle size distribution of 1.12 (D_90_/D_10_). We have investigated a range of different pore-sizes and phases (C_18_, C_4_) for the analysis of a diverse range of nucleic acids including oligonucleotides, oligoribonucleotides, phosphorothioate oligonucleotides and high molecular weight dsDNA and RNA.

## 2 Materials and methods

### 2.1 Chemicals and reagents

Triethylammonium acetate (TEAA, Sigma–Aldrich, UK). Tetrabutylammonium bromide (TBAB, Fluka, UK). Acetonitrile and water (HPLC grade, Fisher Scientific, UK). 1,1,1,3,3,3,-Hexafluoro-2-propanol (HFIP, Sigma–Aldrich, UK). Oligonucleotides were synthesised by Eurofins Scientific (UK). RNase A (Ambion, UK).

### 2.2 IP RP HPLC

Mobile phases were prepared with HPLC grade solvents (Fisher Scientific, UK).

Nucleic acids were analysed by IP RP HPLC on a U3000 RSLC UPLC (Thermo Fisher, UK) with the following columns: Accucore C_18_ 150 mm × 2.1 mm I.D. (2.6 μm superficially porous silica particles 80 Å pore size), Accucore C_18_ 150 mm × 2.1 mm I.D. (2.6 μm superficially porous silica particles 150 Å pore size). The Accucore columns contain 2.6 μm solid-core particles, with a porous layer of 0.5 μm with an average particle size distribution of 1.12 (D_90_/D_10_). Accucore C_4_ 150 mm × 4.6 mm I.D. (a research column based on superficially porous silica 4.0 μm superficially porous silica particles 400 Å pore size). Chromatograms were recorded using a VWD detector at 260 nm with a 2.5 μl flow cell. Tubing was 100 μm I.D. for flow rates of 0.4 ml/min and 180 μm I.D. for flow rates of 1.0 ml/min.

### 2.3 IP RP HPLC mobile phases

Buffer A; 0.1 M triethylammonium acetate (TEAA) pH 7.4. Buffer B; 0.1 M TEAA, pH 7.4, 25% (v/v) acetonitrile. Buffer C; 2.5 mM tetrabutylammonium bromide (TBAB). Buffer D; 2.5 mM tetrabutylammonium bromide, 80% acetonitrile. Buffer E; 20 mM triethylammonium acetate (TEAA) pH 7.4, 80 mM 1,1,1,3,3,3,-hexafluoro-2-propanol (HFIP). Buffer F; 20 mM triethylammonium acetate (TEAA), pH 7.4, 80 mM 1,1,1,3,3,3,-hexafluoro-2-propanol (HFIP) 25% (v/v) acetonitrile. Buffer G; 0.1 M triethylammonium acetate (TEAA) pH 7, 80 mM 1,1,1,3,3,3,-hexafluoro-2-propanol (HFIP). Buffer H; 0.1 M triethylammonium acetate (TEAA), pH 7.4, 80 mM 1,1,1,3,3,3,-hexafluoro-2-propanol (HFIP) 25% (v/v) acetonitrile.

**Gradient 1:** HPLC buffers A and B. Linear gradient: 25–35% B in 1 min; 35–70% B in 14 min; 70–100% B in 0.1 min; 100% B for 3 min; 100–30% B in 0.4 min; 25% B for 5 min. Flow rate: 0.4 ml min^−1^.

**Gradient 2:** HPLC buffers C and D. Linear gradient: 55% B for 1 min; 50–85% B in 14 min; 85–100% B in 0.5 min; 100% B for 3 min; 100–50% B in 1 min; 55% B for 4 min. Flow rate: 0.4 ml min^−1^.

**Gradient 3:** HPLC buffers E and F. Linear gradient 10–30% F in 20 min, 30–90% F in 1 min; 90% F for 3  min; 90–10% F in 0.4 min; 10% F for 5 min at a flow rate of 0.4 ml min^−1^.

**Gradient 4:** HPLC buffers E and F. Linear gradient 10–20% F in 20 min, 20–30% F over 10 min; 30% F to 90%F in 1 min; 90% F for 3 min; 90–10% F in 0.4 min; 10% F for 5 min at a flow rate of 100 μl min^−1^.

**Gradient 5:** HPLC buffers A and B. Linear gradient: 45–50% B in 1 min; 50–80% B in 20 min; 85% B to 100% B in 1 min; 100% B for 2 min; 100–45% B in 0.4 min; 45% B for 4 min. Flow rate: 0.4 ml min^−1^.

**Gradient 6:** HPLC buffers A and B. Linear gradient: 35–40% B in 1 min; 40–60% B in 10 min; 60–68% B in 5 min, 68% B to 100% B in 1 min; 100% B for 2 min; 100–35% B in 0.4 min; 30% B for 4 min. Flow rate: 1.6 ml min^−1^.

**Gradient 7:** HPLC buffers A and B. Linear gradient: 15–60% B in 12 min; 60–100% B in 0.1 min; 100% B for 2 min; 100–15% B in 0.4 min; 15% B for 4 min. Flow rate: 0.4 ml min^−1^.

**Gradient 8:** HPLC buffers A and B. Linear gradient 25–30%B in 1 min; 30–45% B 15 min; 45–100% B in 0.1 min; 100% B for 3 min; 100–25% B in 0.4 min; 25% B for 5 min. Flow rate: 0.4 ml min^−1^.

**Gradient 9:** HPLC buffers A and B. Linear gradient 15–20% B in 1 min; 20–75% B 10 min; 75–100% B in 0.1 min; 100% B for 3 min; 100–15% B in 0.4 min; 25% B for 5 min. Flow rate: 0.4 ml min^−1^.

**Gradient 10:** HPLC buffers E and F. Linear gradient 15–20% F in 1 min; 20–75% F 10 min; 75–100% F in 0.1 min; 100% F for 3 min; 100–15% F in 0.4 min; 25% F for 5 min. Flow rate: 0.4 ml min^−1^.

**Gradient 11:** HPLC buffers C and D. Linear gradient 55–90% D in 15 min; 90–100% D in 0.1 min; 100% D for 3 min; 100–55% D in 0.4 min; 55% D for 5 min. Flow rate: 0.4 ml min^−1^.

**Gradient 12:** HPLC buffers E and F. Linear gradient 35–40% F in 1 min; 40–90% F 10 min; 90–100% F in 0.1 min; 100% F for 3 min; 100–35% F in 0.4 min; 35% F for 5 min. Flow rate: 0.4 ml min^−1^.

**Gradient 13:** HPLC buffers C and D. Linear gradient 55–80% D in 15 min; 85–100% D in 0.1 min; 100% D for 3 min; 100–55% D in 0.4 min; 55% D for 5 min. Flow rate: 0.4 ml min^−1^.

### 2.4 RNase mass mapping

Following purification, 1 μg of ssRNA in RNase-free water was incubated with 1 U RNase A at 37 °C for 30 min. Subsequently, 500 ng, was analysed using LC ESI MS with an Accucore C_18_ column (2.6 μm superficially porous silica particles 80 Å pore size 150 mm × 2.1 mm ID) and gradient 4. Mass spectrometry was performed on a maXis Ultra High Resolution Time of Flight Instrument (Bruker Daltonics) in negative ion mode with a selected mass range of 300–2500 *m*/*z*. An ion source voltage of −2000 V and N_2_ source gas temperature of 300 °C at 6.0 L/h was used to maintain a capillary current of 30–50 nA, with nebuliser gas pressure at 0.4 bar. A list of theoretical monoisotopic masses of oligoribonucleotides was generated using Mongo Oligo Mass Calculator (http://rna.rega.kuleuven.ac.be/masspec/mongo.htm). The output of this software produces a theoretical sequence ladder of oligoribonucleotides for the ssRNA with all possible chemical termini including; 5′-OH, -phosphate, -cyclic phosphate and 3′-OH, -phosphate, -cyclic phosphate.

## 3 Results and discussion

### 3.1 IP RP HPLC analysis of oligonucleotides using superficially porous silica particles

**Fig. 1:**
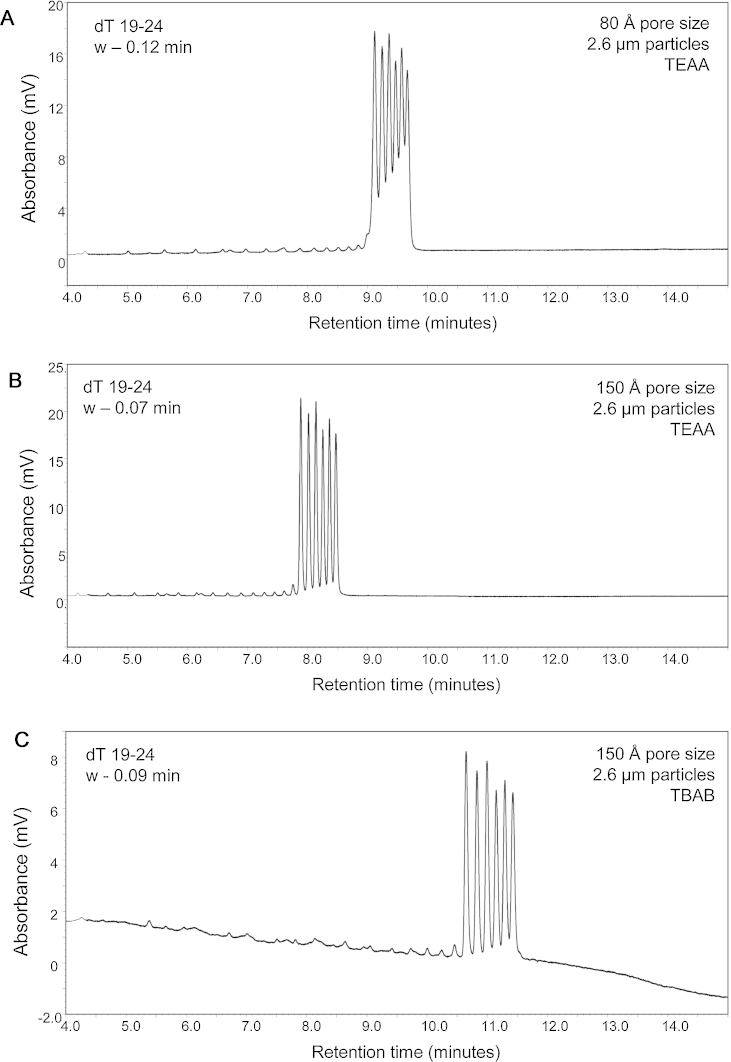
Effect of pore size on the analysis of oligodeoxynucleotides using IP RP HPLC. IP RP HPLC analysis of oligo dT ladder (19–25 nt) on superficially porous particles. (A) 80 Å pore size using TEAA gradient 1. (B) 150 Å pore size using TEAA gradient 1. (C) 150 Å pore size using TBAB gradient 2. 250 ng of oligo dT was analysed at 50 °C, UV detection at 260 nm.

To evaluate the performance and effects of pore size of the solid-core particles for the analysis of oligonucleotides, a 2′-deoxythymine ladder (dT 19–24) was analysed by IP RP HPLC with triethylammnonium acetate (TEAA) as the ion pair reagent in conjunction with C_18_ 2.6 μm solid-core particles with 80 Å and 150 Å pore sizes (see Fig. 1A and B). The results show that the pore size of the solid-core particles affects the resolution of the dT ladder. Typical peak widths of 0.12 min were obtained on the 80 Å compared to 0.07 min on the 150 Å pore size solid-core particles, demonstrating that increased resolution of the oligonucleotides were obtained on the 150 Å pore size solid-core particles at a flow rate of 0.4 ml/min. The 80 Å shows restricted diffusion for the oligonucleotides under these conditions, resulting in broader peaks in comparison to the 150 Å pore size solid-core particles. These data are consistent with those observed for the analysis of peptides, where increased resolution is observed on the wider pore sized particles [29]. Further application using the 150 Å pore size solid-core particles was also examined using an alternative ion pair reagent tetrabutylammonium bromide (TBAB) which enables size-dependent separations of the oligonucleotides (see Fig. 1C). Similar resolution was obtained to the weak ion pair reagent (TEAA) with the same column dimensions. The results of the oligonucleotide separations using the 150 Å pore size solid-core particles demonstrate that rapid, high resolution separation of oligonucleotides can be achieved in a similar way to those obtained using non-porous particles and UPLC applications.

**Fig. 2:**
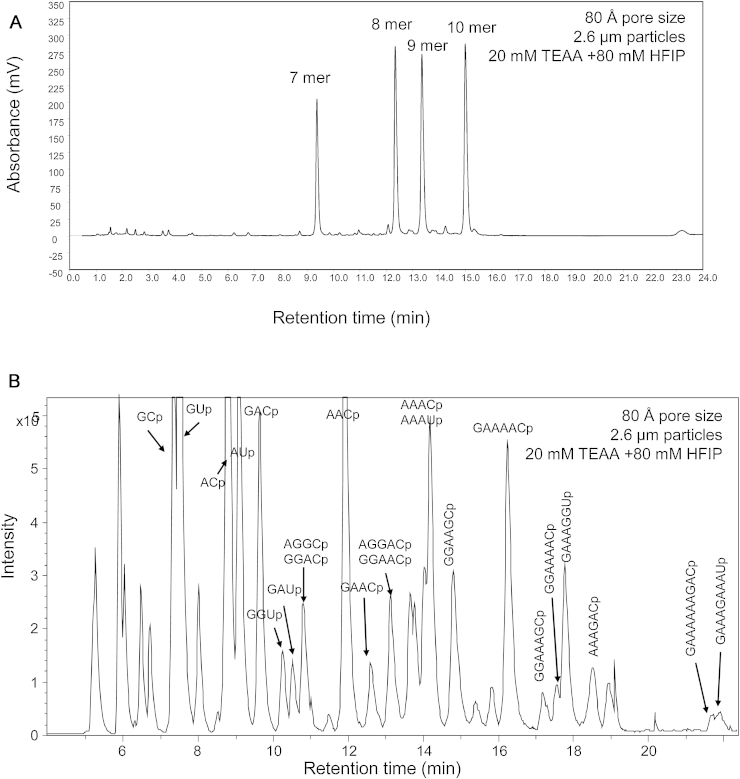
RNase mass mapping using 80 Å pore size superficially porous particles. (A) IP RP HPLC analysis of 7–10 mer oligonucleotides on superficially porous particles, 80 Å pore size using gradient 3. (B) LS ESI MS analysis of RNase a mass mapping of 500 nt ssRNA *in vitro* transcript. 500 ng of ssRNA was analysed using gradient 4. The identified oligoribonucleotides are highlighted.

Further analysis of smaller oligonucleotides (7–10 mers) revealed that improved resolution was obtained on the 80 Å pore sizes in comparison to the 150 Å pore size particles, in contrast to that observed for the larger oligonucleotides (19–24 mers). Analysis of the 7–10 mers was performed using the MS-compatible ion pair solvents (20 mM TEAA + 80 mM HFIP) on the 80 Å column is shown in Fig. 2. The separation of small oligoribonucleotides is required for RNase mapping experiments, where large RNA biomolecules are digested with an RNAse prior to liquid chromatography, often interfaced with mass spectrometry analysis [33]. The application of C_18_ 2.6 μm solid-core particles with 80 Å pore sizes was therefore applied to RNase mapping experiments. A 500 nt ssRNA generated *via in vitro* transcription was digested using RNase A and subsequently analysed using IP RP HPLC interfaced with MS (see Fig. 2B). Oligoribonucleotides were identified by comparing their observed and theoretical monoisotopic masses after an *in silico* RNase A digest and are highlighted in Fig. 2B.

### 3.2 IP RP HPLC analysis of large dsDNA/RNA fragments using superficially porous silica particles

**Fig. 3:**
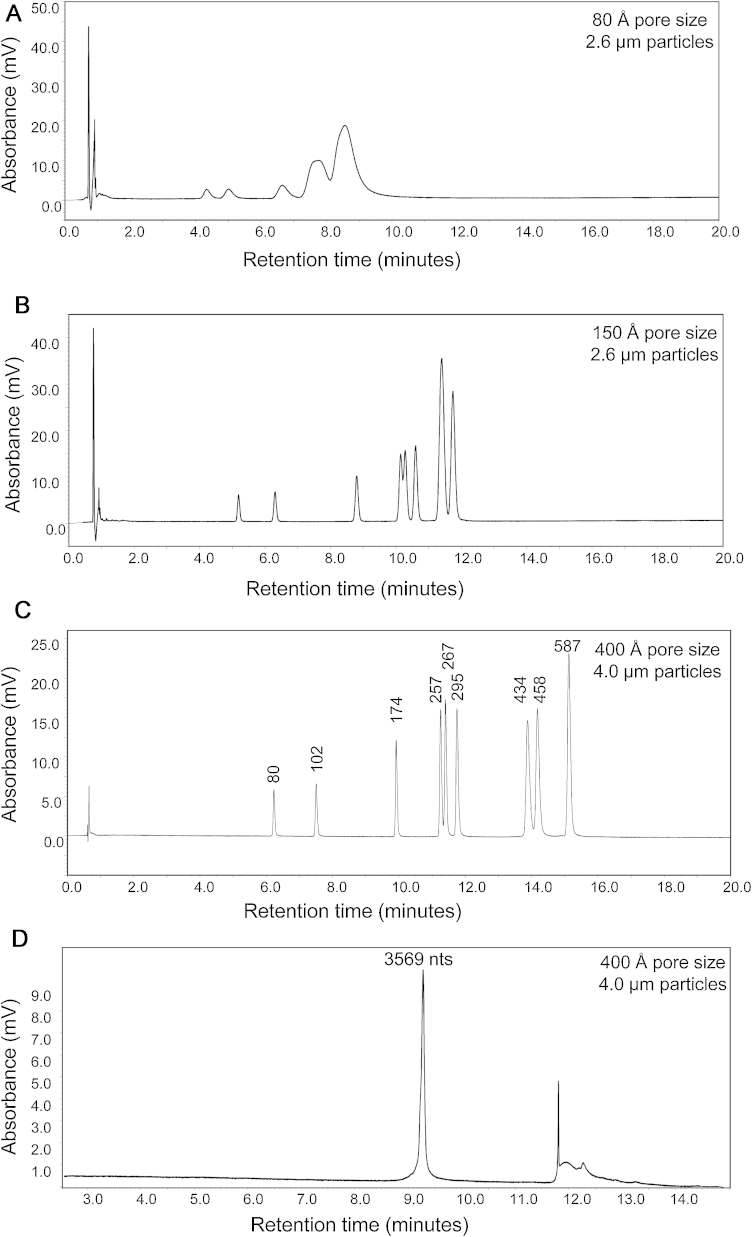
Effect of pore size on the analysis of dsDNA/RNA using IP RP HPLC. IP RP HPLC chromatograms of the analysis pUC18 HaeIII digest analysed using superficially porous particles. (A) 80 Å pore size using TEAA gradient 5. (B) 150 Å pore size using TEAA gradient 5 and (C) 400 Å pore size using TEAA gradient 6. 250 ng of pUC18 HaeIII digest was analysed at 50 °C, UV detection at 260 nm. (D) IP RP HPLC chromatogram of the MS2 RNA on 400 Å pore size superficially porous particles using TEAA gradient 7.50 ng MS2 RNA was injected and analysed at 50 °C UV detection at 260 nm.

To evaluate the separation of dsDNA using IP RP HPLC in conjunction with superficially porous silica particles, a pUC18HaeIII digest, consisting of a range of dsDNA fragments (80–587 bp), was analysed. Chromatograms of the dsDNA fragments on the 80 Å and 150 Å, 2.6 μm superficially porous silica particles is shown in Fig. 3A/B. The results clearly show a significant improvement in the resolution of the dsDNA fragments on the 150 Å pore size solid-core particles in comparison to the 80 Å particles, demonstrating that restricted diffusion for larger *M*_W_ dsDNA fragments leads to broader peaks. Furthermore, the restricted diffusion is more significant for the larger *M*_W_ dsDNA fragments compared to the smaller *M*_W_ oligonucleotides previously analysed using the 80 Å particles. However, the separation efficiency achieved on the 150 Å pore size solid-core particles is not as high as that typically achieved on non-porous 2 mm PS-DVB particles [3], [7]. Therefore, in an approach to further optimise the separation of the larger dsDNA fragments and limit the restricted diffusion observed on both the 80 Å and 150 Å particles, a research column based on superficially porous silica (4.0 mm superficially porous silica particles with 400 Å pore sizes) was evaluated (see Fig. 3C). The chromatogram shows a significant enhancement in the separation of the dsDNA, achieving near baseline separation of the 257/267 bp and 434/458 bp fragments. This observation is consistent with previous findings that increasing the pore size (400 Å) results in improved separation of large intact proteins compared to the 80 Å and 150 Å particles [29]. However, it should be noted that, although the separation of the larger dsDNA fragments is improved using the 400 Å pore size particles, analysis of the oligonucleotides (dT 19–24) revealed a loss in resolution compared to the 150 Å/80 Å particles (data not shown).

The requirement for high throughput analytical tools that can readily separate, purify and analyse ribonucleic acids (RNA) are assuming increasing significance with the recent discoveries of the diverse and important roles RNA plays in biological systems. In particular, the ability to separate and analyse large *M*_W_ RNAs is challenging. However, Fig. 3D shows that the large 3569 nt RNA from bacteriophage MS2 elutes as a narrow peak, demonstrating the high resolution separation of RNA, similar to larger dsDNA fragments, by rapid analysis on 4.0 mm superficially porous silica particles of 400 Å pore size.

Using stationary phases with different pore sizes will alter the available surface area and therefore affect the overall capacity of the column, retention times and possibly the effective gradient [34]. Fig. 1, Fig. 2, Fig. 3 demonstrate the importance of selecting an appropriate sorbent pore size for the analytes of interest.

### 3.3 IP RP HPLC analysis of therapeutic oligonucleotides using superficially porous silica particles

**Fig. 4:**
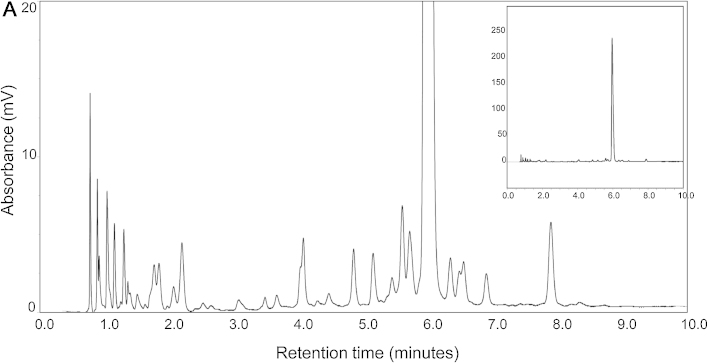
IP RP HPLC analysis of a synthetic oligoribonucleotide using superficially porous particles. 163 pmol of synthetic oligoribonucleotide was analysed at 50 °C, UV detection at 260 nm using gradient 8.

With the emergence of therapeutic oligonucleotides, including siRNA therapeutics, RNA aptamers and antisense oligonucleotides which are all chemically synthesized, the requirement for high throughput, robust approaches to isolate the therapeutic oligonucleotide from impurities such as failure sequences (typically n-1/n-2, also termed short-mers) and long-mers (typically containing deprotecting groups) is an important analytical challenge. The analysis of a typical synthetic oligonucleotide is shown in Fig. 4, demonstrating the ability to resolve the full length product (FLP) from closely related impurities using 2.6 μm solid-core particles with 150 Å pore sizes. Therapeutic oligonucleotides are often synthesised to incorporate diverse chemical modifications to confer stability *in vivo.* These modifications include 2′-*O*-methyl (2′-*O*-Me), *O*-ethyl (2′-*O*-ethyl), *O*-methoxyethyl (2′-MOE), and fluorine (2′-F) substitutions as well as locked nucleic acids (LNA) and phosphorothioate backbone modifications. This synthesis strategy can result in even more complex impurities, including diastereoisomers. The separation of phosphorothioate containing oligonucleotides presents additional challenges due to the presence of a large number of diastereoisomers that often causes peak broadening and poor resolution of closely related impurities. The application of IP RP HPLC for the analysis of fully phosphorothioate containing oligonucleotides has been previously studied using TEA/HFIP (typically 16 mM TEA/400 mM HFIP) in conjunction with porous C_18_ particles. Under these optimised IP conditions, predominantly size-based separations are performed where the peak broadening effect of the different hydrophobicities of phosphorothioate diastereoisomers is eliminated [35], [36], [37]. Further studies using cyclohexyldimethylammonium acetate (CycHDMAA) in conjunction with monolithic, poly(styrene-divinylbenzene)-based capillary columns have also been used to analyse fully phosphorothioate containing oligonucleotides [38].

**Fig. 5:**
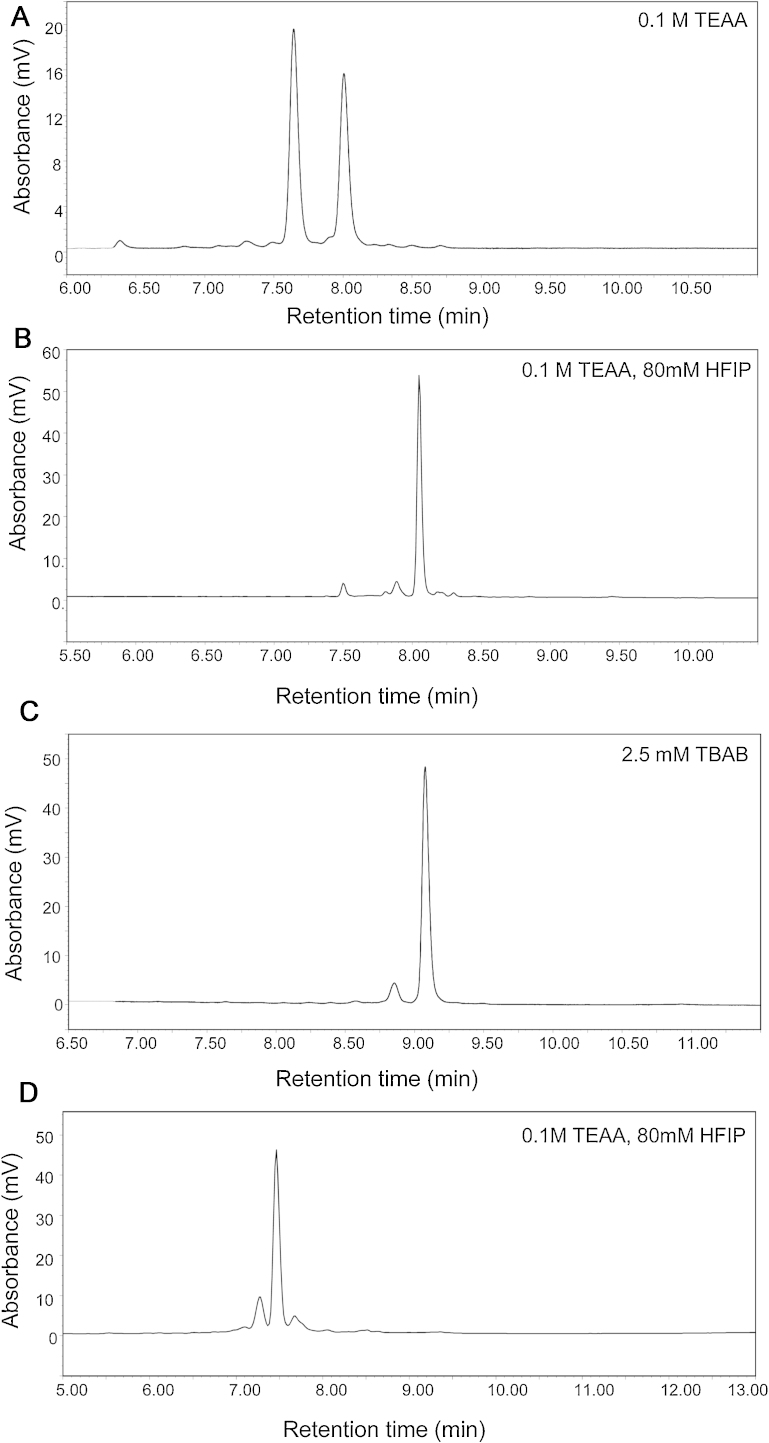
IP RP HPLC analysis of oligonucleotide containing a single phosphorothioate using superficially porous particles 150 Å pore size. (A) Separation of oligonucleotide phosphorothioate diastereoisomers using IP RP HPLC using the weak ion pair reagent TEAA using gradient 9. (B) IP RP HPLC chromatogram of oligonucleotide phosphorothioate diastereoisomers using IP RHPLC using the weak ion pair reagent TEAA in conjunction with HFIP using gradient 10. (C) IP RP HPLC chromatogram of oligonucleotide phosphorothioate diastereoisomers using the strong ion pair reagent TBAB using gradient 11. (D**)** IP RP HPLC chromatogram of a fully phosphorothioated oligonucleotide using the strong ion pair reagent TBAB using gradient 12.60 pmol of each oligonucleotide was injected and analysed at 50 °C, UV detection at 260 nm.

The analysis of an oligonucleotide containing a single phosphorothioate, using 150 Å pore size solid-core particles in conjunction with the weak ion pairing reagent TEAA is shown in Fig. 5A. Short alkyl chains such as those in TEAA, allow the stationary phase to partially retain its hydrophobic or reverse phase properties as TEAA only partially covers the stationary phase. Therefore, separation is dependent on both size and base composition. The results demonstrate the ability to rapidly separate the two diastereoisomers (*R*_P_ and *S*_P_) that are present similar to previous studies utilising alternative stationary phases [39], [40], [41]. However, analysis of fully phosphorothioate containing oligonucleotides under weak ion pair reagent conditions leads to the appearance of a broad peak due to the presence multiple diastereoisomers which are likely to co-elute with various shortmer and longmer impurities. To promote the co-elution of the phosphorothioate diastereoisomers and therefore reduce the peak width of fully phosphorothioate containing oligonucleotides, the analysis was performed in the presence of HFIP and alternatively using strong ion pair reagent tetrabutylammonium bromide (TBAB). Using TBAB the longer alkyl chains provide complete coverage of the stationary phase and a dynamic anion-exchange is the predominant mechanism, resulting in size based separation with limited sequence effects. Analysis under these conditions promotes size dependent separations and minimises the effects of the diastereoisomers, as shown in Fig. 5B and C. Under these conditions the two diastereoisomers of the singly phosphorothioated oligonucleotide co-elute and as expected the peak width of the fully phoshorothioated oligonucleotide is significantly reduced (see Fig. 5D). Although utilising the ion pair reagent TBAB and the addition of HFIP minimises the effects of the diastereoisomers in fully phosphorothioate containing oligonucleotides, their analysis still remains challenging as the effects are not fully supressed. This can be seen by comparing the peak width of the fully phosphorothioate oligonucleotide (0.17 min) and fully phosphodiester oligonucleotide (0.11 min) of the same sequence analysed on the same gradient slope (see Supplementary Fig. 1).

**Fig. 6:**
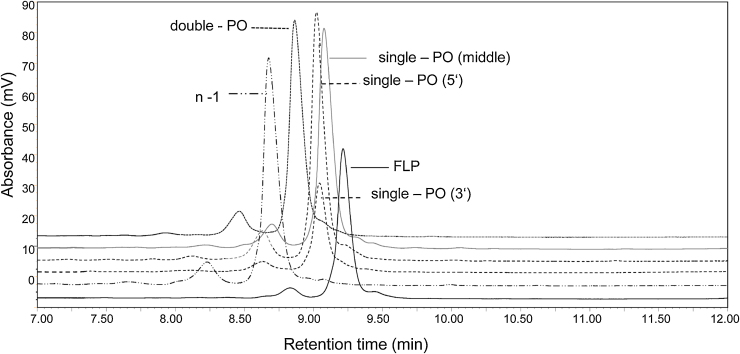
IP RP HPLC analysis of fully phosphorothioated oligonucleotide and related impurities using superficially porous particles 150 Å pore size. Overlay of fully phosphorothioated oligonucleotide, fully phosphorothioated n-1, single phosphodiester 5′-end, single phosphodiester 3′-end, single phosphodiester middle, double phosphodiester 5′/3′-ends. 80 pmol of each oligonucleotide was injected and analysed at 50 °C using the strong ion pair reagent TBAB gradient 13 with UV detection at 260 nm.

In addition, the ability to separate typical impurities generated during the synthesis of a fully phosphorothioated oligonucleotide was also studied. A number of common impurities including the oxidised phosphodiester were synthesised to represent common contaminants (see Table 1). To evaluate the ability of the superficially porous silica particles to separate the single phosphodiester containing oligonucleotide (5′-end, 3′-end and the middle), double phosphodiester oligonucleotide and n-1 truncated oligonucleotide from the full length fully phosphorothioate oligonucleotide, the analysis was performed using the previously optimised conditions with the strong ion pair reagent TBAB (see Fig. 6). The results demonstrate the separation of the full length fully phosphorothioate oligonucleotide from the single/double phosphodiester impurities and the n-1 truncated species. The ability to resolve these common impurities on superficially porous silica particles with 150 Å pore size in conjunction with optimised sequence dependent separations and cost effective solvents, including low amounts of HFIP to TEAA and strong ion pair reagents such as TBAB, provides a significant development in the analysis of therapeutic oligonucleotides including fully phosphorothioate oligonucleotides.

## 4 Conclusions

Superficially porous silica particles enable the rapid, high resolution separation of nucleic acids using IP RP HPLC. The pore sizes of the C_18_ superficially porous silica particles significantly effects the resolution of the nucleic acids. Optimum separations of small oligonucleotides such as those generated in RNase mapping experiments were obtained with 80 Å pore sizes and can readily be interfaced with mass spectrometry analysis. Improved resolution of larger oligonucleotides (>19 mers) was observed with pore sizes of 150 Å. The optimum resolution for larger dsDNA/RNA molecules was achieved using superficially porous silica particles with pore sizes of 400 Å. Therefore, it is important to select the appropriate pore sizes of the C_18_ superficially porous silica particles prior to analysis of nucleic acids.

The analysis of an oligonucleotide containing a single phosphorothioate backbone modification, using 150 Å pore size solid-core particles in conjunction with the weak ion pairing reagent TEAA enabled the separation of the two diastereoisomers (*R*_P_ and *S*_P_) that are present. The use of superficially porous silica particles in conjunction with strong ion pair reagents (TBAB) promotes size dependent separations and was shown to minimise the effects of the diastereoisomers. In addition, the ability to separate typical impurities generated during the synthesis of a fully phosphorothioated oligonucleotide using superficially porous silica particles (150 Å pore size) was shown in conjunction with optimised sequence dependent separations. The results demonstrate the ability to separate the full length fully phosphorothioate oligonucleotide from the single/double phosphodiester impurities and the n-1 truncated species which are often generated in the synthesis of this important class of therapeutic oligonucleotide.

## Figures and Tables

**Table 1:** Oligonucleotide sequences used to represent the fully phosphorothioated oligonucleotide and common related impurities, po indicates phosphodiester bond.

Oligonucleotide	Sequence 5′-3′
Fully phosphorothioated	ACAAAAGTCCGTGAG
Single phosphodiester 5′	ApoCAAAAGTCCGTGAG
Single phosphodiester 3′	ACAAAAGTCCGTGApoG
Single phosphodiester middle	ACAAAAGpoTCCGTGAG
Double phosphodiester	ApoCAAAAGTCCGTGApoG
n-1	CAAAAGTCCGTGAG
