# Supplementary material for: Nucleic acid separations using superficially porous silica particles
Source: J Chromatogr A. 2016 Apr 1;1440:135–44. doi: 10.1016/j.chroma.2016.02.057 (PMC4801196; doi:10.1016/j.chroma.2016.02.057)
Supplement: Supplementary file 1 [file mmc1.pdf]

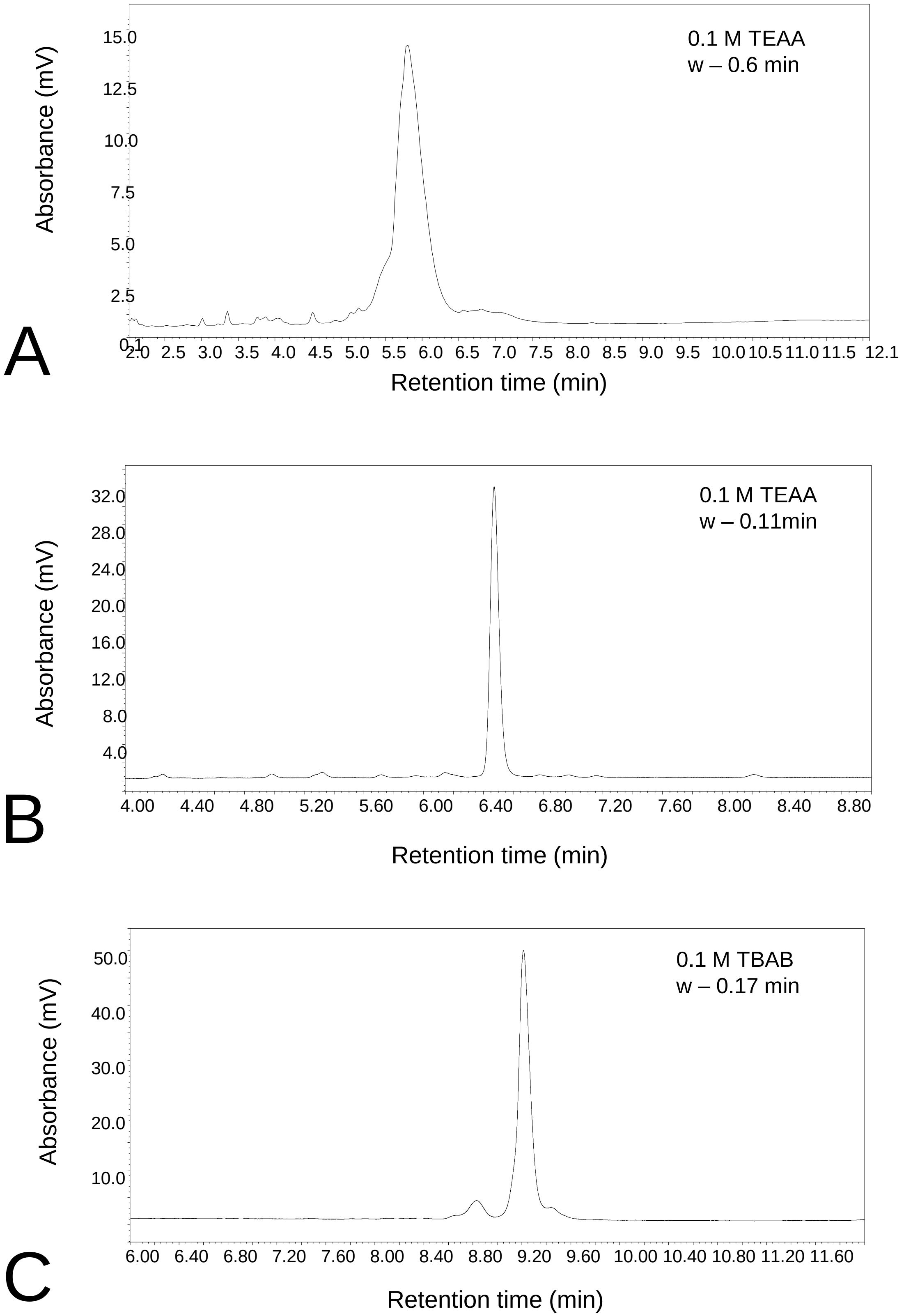

Supplementary Figure 1. IP RP HPLC analysis of fully phosphorothioate oligonucleotide and a fully phosphodiester oligonucleotide using superficially porous particles 150 Å pore size. **A**) IP RP HPLC chromatogram of a fully phosphorothioate oligonucleotide using TEAA. HPLC buffers A and B. Linear gradient: 45 – 50% B in 1 min; 50 – 80% B in 14 min; 80 – 100% B in 0.1 min; 100% B for 3 min; 100 – 45% B in 0.4 min; 45% B for 5 min. Flow rate: 0.4 ml min^{-1} **B**) IP RP HPLC chromatogram of fully phosphodiester oligonucleotide using TEAA . HPLC buffers A and B. Linear gradient: 15 – 20% B in 1 min; 20 – 45% B in 14 min; 45 – 100% B in 0.1 min; 100% B for 3 min; 100 – 15% B in 0.4 min; 15% B for 5 min. Flow rate: 0.4 ml min^{-1} **C)** IP RP HPLC chromatogram of fully phosphorothioate oligonucleotide using TBAB. HPLC buffers C and D. Linear gradient: 50 – 55% D in 1 min; 55 – 80% D in 14 min; 80 – 100% D in 0.1 min; 100% D for 3 min; 100 – 50% D in 0.4 min; 50% D for 5 min. Flow rate: 0.4 ml min^{-1}. 60 pmoles of each oligonucleotide corresponding to the sequence 5’-ACAAAAGTCCGTGAG-3’ was analysed at 50 °C, UV detection at 260 nm.
